# Impact of daily versus weekly service of infectious diseases consultation on hospital antimicrobial consumption: a retrospective study

**DOI:** 10.1186/s12879-020-05550-1

**Published:** 2020-11-07

**Authors:** Andrea Cona, Lidia Gazzola, Ottavia Viganò, Teresa Bini, Giulia Carla Marchetti, Antonella d’Arminio Monforte

**Affiliations:** Clinic of Infectious and Tropical Diseases, Department of Health Sciences, ASST Santi Paolo e Carlo, University of Milan, Via A. di Rudinì 8, 20142 Milan, Italy

**Keywords:** Infectious diseases consultant, Antibiotic consumption, Antimicrobial stewardship

## Abstract

**Background:**

To verify whether a daily service of Infectious Diseases consultation (ID-cons) is more effective than a weekly service in reducing antibiotic (ATB) consumption without worsening of clinical outcomes.

**Methods:**

Two-year observational analysis of the ID-cons provided in a hospital setting in Milan, Italy. ID-cons resulted in: start-of-ATB; no-ATB; confirmation; modification-of-ATB. The impact of a weekly (September 1, 2016 - August 31, 2017 versus a daily (September 1, 2017 – September 30, 2018) service of ID-cons was evaluated in terms of: time-from-admission-to-first-ID-cons, type of ATB-intervention and number-of-ID-cons per 100 bed-days (bd). Primary outcomes: reduction of hospital ATB consumption overall and by department and classes expressed as Defined Daily Dose (DDD)/100bd (by Wilcoxon test for paired data). Secondary outcomes: overall and sepsis-related in-hospital annual mortality rates (as death/patient’s admissions).

**Results:**

Overall 2552 ID-cons in 1111 patients (mean, 2.3 ID-cons per patient) were performed (18.6% weekly vs 81.4% daily). No differences in patient characteristics were observed. In the daily-service, compared to the weekly-service, patients were seen by the ID-consultant earlier (time-from-admission-to-ID-cons: 6 days (IQR 2–13) vs 10 days (IQR 6–19), *p* < 0.001) and ATB was more often started by the ID-consultant (Start-of-ATB: 11.6% vs 8%, *p* = 0.02), rather than treating physicians. After switching to daily-service, the number-of-ID-cons increased from 0.4/100bd to 1.5/100bd (*p* = 0.01), with the greatest increase in the emergency department (1.5/100bd vs 6.7/100bd, *p* < 0.001). Total ATB consumption decreased from 64 to 60 DDD/100bd. As for the number-of-cons, the consumption of ATB decreased mainly in the emergency area. According to ATB classes, glycopeptides consumption was reduced from 3.1 to 2.1 DDD/100bd (*p* = 0.02) while carbapenem use decreased from 3.7 to 3.1 DDD/100bd (*p* = 0.07). No changes in overall mortality (5.2% vs 5.2%) and sepsis-related mortality (19.3% vs 20.9%; *p* = 0.7) were observed among the two time-period.

**Conclusions:**

Daily-ID-cons resulted in a more comprehensive management of the infected patient by the ID-consultant, especially in the emergency area where we also observed the highest rate of reduction of ATB-usage. No change in mortality was observed.

## Background

The development and spread of multidrug-resistant bacteria (MDR) is mainly due to the misuse/overuse of antimicrobials and to the lack of effective infection control measures [1]. Antimicrobial resistance (AMR) leads to infections which are more difficult to treat and to an increased number of inappropriate antimicrobial prescriptions [2]**.** AMR and subsequent inappropriate antimicrobial therapy increase morbidity, duration of hospital stay and mortality [3–5]. In a meta-analysis, Marquet et al. [5], reported 30-day and in-hospital increases mortality in patients receiving inappropriate therapy. Other studies found better patient-related outcomes, such as reduced 30-day mortality and duration of hospital stay, when appropriate antimicrobial therapy was prescribed [6–10]**.** Given the impact of adequate antibiotic treatment on patient outcomes, the infectious diseases specialist represents an added value, as suggested by several studies showing the positive effect of infectious diseases consultation [11–15]. Nonetheless, little is known on the impact of infectious diseases consultations on the consumption of antibiotics.

To bridge this gap, we aimed to verify whether a daily service of Infectious Diseases consultation (ID-cons) is more effective than a weekly service in reducing antibiotic consumption without worsening of clinical outcomes.

## Methods

### Design and study setting

The study consists in a two-year retrospective observational analysis of all the ID-cons provided in a large tertiary hospital in Milan, Italy. San Carlo Borromeo Hospital (HSC) is a non-teaching public hospital with 494 beds and 20,000 admissions/year. HSC does not feature a transplant or haematology-oncology unit but does have a neurosurgery unit. Thus, possible source and aetiology of infections might account for these characteristics. Additionally, HSC does not have an ID-unit but rather a consult service that is staffed by ID-specialists from the Unit of Infectious Diseases of San Paolo Hospital since 2016.

Infectious Diseases consultations were provided once a week from September 1, 2016 to August 31, 2017 (weekly ID-cons period); from September 1, 2017 onwards the service of ID-consultation is provided on a daily basis (daily ID-cons period). All the ID-cons were performed by the same team of ID-consultants. ID-cons is defined as any request by the treating non-ID physician for ID advice with bed-side evaluation of the patient resulting in a written statement by the ID consultant.

### Study procedures and definitions

Demographics, clinical conditions and microbiological findings of all hospitalized patients for whom an ID consultation was required from September 1, 2016 to September 30, 2018 were collected in a dedicated database. Clinical charts were reviewed to identify risk factors of infections (alcoholism, radio-chemotherapy, use of steroids, injection drug use), and presence of comorbidities (cardiovascular disease, dementia, liver cirrhosis, cancer, chronic obstructive pulmonary disease, chronic renal failure, diabetes, hepatitis C virus (HCV) infection). Comorbidities were evaluated according to the age-adjusted Charlson comorbidity index (ACCI), a validated prognostic tool that predicts the risk of death in patients with several comorbidities [16].

Hospital units were grouped into three departments:

- Medical Department, which includes: Cardiology, Gastroenterology, Internal Medicine Units, Oncology, Nephrology, Neurology, Pulmonary, Psychiatry and Rehabilitation Unit;
- Surgical Department: General Surgery, Neurosurgery, Obstetrics/gynecology, Orthopedic Unit, Urology and Vascular Surgery;
- Emergency Department: Coronary Unit, Intensive care Unit (ICU) and Sub-intensive care Units, Emergency Room, Emergency Medicine Unit and Stroke Unit.

Infections were classified as: 1) healthcare-associated infections (HAI), in the case of infections associated with hospitalization or other medical treatment that appeared 48 h or more after hospital admission; 2) community-acquired infections (CAI), in the case of patients admitted for an infection acquired before hospital admission or diagnosed within 48 h of admission [17]. Classification of an infection into one of the two groups, HAI or CAI, was made by the ID consultant by combining clinical presentation with radiological and microbiological findings.

At each consultation all the antimicrobial therapies were reviewed and discussed with the treating physician. Both intravenous and oral antimicrobials were included in the study.

Interventions on antibiotic therapies were collected into the database according to the following classification: start of ATB, no need of ATB, confirmation of ATB and modification of ATB (including dosage optimization, change of ATB, de-escalation, intensification and discontinuation of ATB). De-escalation therapy was defined as: i) switching from combination to monotherapy; ii) narrowing spectrum of activity. An opposite definition was applied to intensification therapy.

Treatment was considered appropriate in case of “confirmation of ATB” by the ID consultant in terms of dose, duration, penetrability and choice of regimen. In case of microbiological findings, appropriateness was assessed based on in vitro susceptibility data. Assessment of appropriateness was performed on the basis of internal guidelines that refer, in turn, to national and international guidelines. Conversely, “modification of ATB” was considered a marker of inappropriate therapy. In order to evaluate the appropriateness of antibiotics prescribed by the non-ID specialist physician, only first consultations per patient with an already ongoing antibiotic therapy were analyzed.

### Inclusion/exclusion criteria and study period

All hospitalized patients for whom the treating physician required an ID consultation, from September 1, 2016 to September 30, 2018 were included in the study. All the ID-cons, both first-time and follow-up visits, were included. Age < 18 years was the only criteria for exclusion.

We evaluated the impact of a weekly service of ID-cons (September 1, 2016 - August 31, 2017) versus a daily service of ID-cons (September 1, 2017 – September 30, 2018).

### Outcomes

Process outcomes estimate the performance of the study. Process outcomes of the study are: i) number-of-ID-cons per 100 bed-days (bd), ii) days-from-admission-to-first-ID-cons, iii) type of ATB -intervention and iv) appropriateness of ATB prescription (evaluated only on first ID evaluation).

Primary outcomes of the study are: (i) the reduction of overall ATB consumption and (ii) the reduction of ATB consumption by department and by ATB classes expressed as Defined Daily Dose (DDD)/100bd.

The secondary outcomes were the overall and sepsis-related in-hospital annual mortality rates (as death/patient’s admissions) evaluated from January 1st, 2017 to December 31st, 2018.

### Statistical analysis

Categorical variables were analysed using absolute numbers and percentages while continuous variables were analysed using the median and interquartile range (IQR). Chi-square test was used for sex, comorbidities and characteristics of the infections while Mann-Whitney U test was used for age and ACCI. ATB consumption was expressed as DDD/100bd. The indicator 100 bed-days is widely applied in antimicrobial stewardship programs for analyses of in-hospital drug use according to World Health Organization (WHO) recommendations. DDD, defined as “the assumed average maintenance dose per day for a drug used for its main indication in adults”, were calculated using the Anatomical Therapeutic Chemical/DDD Index 2020 of the WHO Center for Drugs Statistics Methodology [18]. In order to evaluate the impact of our intervention on the outcome, a sensitivity analysis including units with high number of ID cons/100bd (≥ 25th percentile of the ID-cons distribution) was performed. Differences in patient characteristics and process outcomes were evaluated by Mann-Whitney U and Chi-square tests. All comparisons were performed by time-period (before and after the implementation of daily ID-cons). Differences in antimicrobial consumption between the two time periods were evaluated by Wilcoxon test for paired data considering ATB consumption in every single unit. A *p*-value < 0.05 was considered statistically significant. Statistical analyses were performed with SAS software (version 9.2).

## Results

### Patient characteristics

Overall, 2552 ID-cons were performed in 1111 patients with a mean of 2.3 ID-cons per patient, including follow-up visits. ID-cons were distributed among the two time periods as follows: 18.6% (475/2552) in the weekly period versus 81.4% (2077/2552) in the daily period.

The 1111 patients included in the study were distributed as follows: 24.6% (273/1111) in weekly ID-cons and 75.4% (838/1111) in daily ID-cons.

An increment in the number of follow-up visits was observed switching from the weekly to the daily period. In fact, the mean of ID-cons per patient increased from 1.7 to 2.5.

Demographics and baseline characteristics of the study population are shown in Table 1. Approximately 40% of the population was female and the median age was 73.5 (IQR 61–81) years. Median ACCI was 6 (IQR 4–8). The most common comorbidity was cardiovascular disease, followed by diabetes mellitus and neoplastic diseases. The patients in the two groups, weekly ID-cons and daily ID-cons, were comparable for sex, age and comorbidities.

**Table 1:** Demographics and baseline characteristics of the study population

	Total patients *n* = 1111	Weekly period *n* = 273	Daily period *n* = 838	*p value*
Female sex, %	460 (41.4%)	115 (42%)	345 (41%)	.7
Age, median (IQR)	73.5 (61–81)	72 (61–81)	75 (61–82)	.1
ACCI, median (IQR)	6 (4–8)	6 (4–8)	6 (4–8)	.2
Comorbidities, n (%):				
Chronic Renal Failure	166 (14.9%)	36 (13.2%)	130 (15.5%)	.8
Cardiovascular disease	549 (49.4%)	125 (45.8%)	424 (50.6%)	.14
Diabetes mellitus	227 (20.4%)	51 (18.7%)	176 (21%)	.6
Dementia	122 (11%)	15 (5.5%)	107 (12.8%)	.07
Liver cirrhosis	89 (8%)	18 (6.6%)	71 (8.5%)	.6
Cancer	185 (16.7%)	42 (15.4%)	143 (17.1%)	.1
COPD	156 (14%)	36 (13.2%)	120 (14.3%)	.6

Data are presented as n (%) or median (IQR); *IQR* Interquartile range, *COPD* Chronic obstructive pulmonary disease

Characteristics and distribution of the infections are shown in Table 2.

**Table 2:** Characteristics and distribution of the infections

	Total patients ***n*** = 1111	Weekly period ***n*** = 273	Daily period ***n*** = 838	***p value***
HAIs	575 (51.8%)	118 (43.2%)	457 (54.5%)	.001
CAIs	536 (48.2%)	155 (56.8%)	381 (45.5%)	
Site of Infection:				
UTIs	126 (11.3%)	10 (3.8%)	116 (14.4%)	< 0.001
Cardiovascular	32 (2.9%)	11 (4.1%)	21 (2.6%)	.19
Bone and joint	66 (5.9%)	20 (7.6%)	46 (5.7%)	.26
ABSSSIs	74 (6.7%)	17 (6.4%)	57 (7.1%)	.74
Intra abdominal	138 (12.4%)	35 (13%)	103 (12.9%)	.81
Pneumonia	220 (19.8%)	46 (17.5%)	174 (21%)	.15
CNS	29 (2.6%)	13 (4.9%)	16 (2.0%)	.01
Sepsis	193 (17.3%)	40 (15.2%)	153 (19.1%)	.17
Other	182 (16.4%)	70 (26%)	112 (14%)	< 0.001

Data are presented as n (%); *HAIs* Healthcare-associated infections, *CAIs* Community-acquired infections, *UTIs* Urinary tract infections, *ABSSSIs* Acute Bacterial Skin and Skin Structure Infections, *CNS* Central Nervous System

Just over half of the infections (575/1111) evaluated during consultancies were healthcare-associated.

Overall, the most represented infection was pneumonia, followed by sepsis. Urinary tract infections (UTIs) and sepsis were more represented in the daily ID-cons period (UTIs 15% vs 4; sepsis 19% vs 15%) while central nervous system (CNS) infections and bone and joint infections were more common in the weekly ID-cons period (CNS infections 5% vs 2%; bone and joint infections 8% vs 6%) (Table 2).

### Process outcomes

Four process outcomes were evaluated in the study: i) number-of-ID-cons per 100bd, ii) days-from-admission-to-first-ID-cons, iii) type of ATB intervention and iv) appropriateness of ATB prescription (evaluated only on first ID evaluation).

**Fig. 1:**
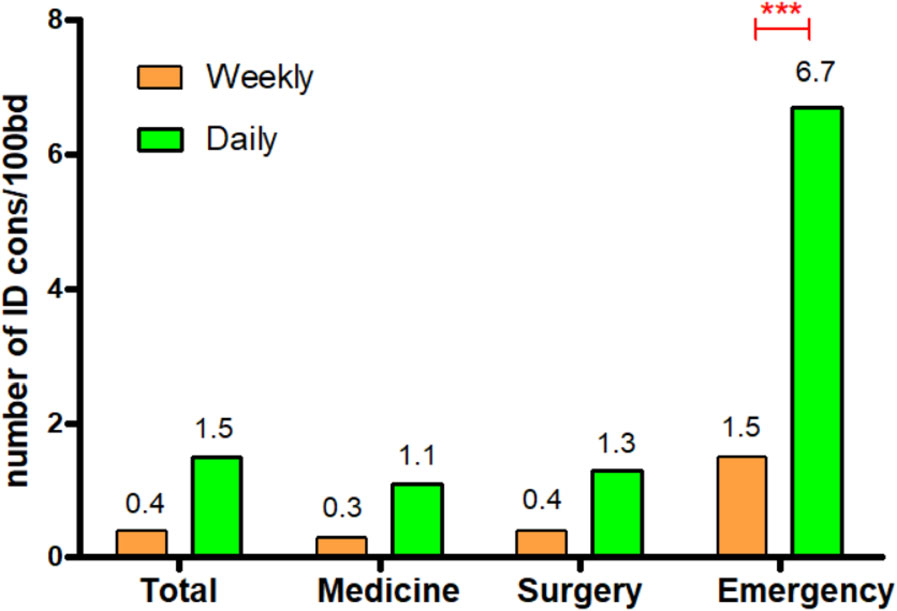
Number of ID-cons per 100 bed-days performed over the study period. *BD* bed-days

As expected, switching from weekly to daily service the number of ID-cons performed significantly increased. In fact, the number-of-ID-cons/100bd increased from 0.4 to 1.5 (*p* = 0.01) in the whole hospital. Analyzing the data by department, the greatest increase in the number of ID-cons was observed in the emergency department (from 1.5 to 6.7 number-of-ID-cons/100bd, *p* < 0.001) (Fig. 1). Focusing only in ICU, ID-cons/100bd increased from 2 to 5 (*p* < 0.001).

In the daily service, patients were seen by the ID specialist earlier. In fact, days-from-admission-to-first-ID-cons decreased from 10 days in the weekly service (IQR 6–19) to 6 days in the daily service (IQR 2–13; *p* < 0.001). Furthermore, antibiotic therapy was more often started by the ID consultant (start of ATB: 11.6% (38/475) vs 8% (242/2077), *p* = 0.02).

As previously stated, appropriateness was evaluated including only the first ID-cons per patient with anATB prescribed by the non-ID treating physician. Following switch to the weekly service of ID-cons, appropriateness rose from 26 to 34% (weekly 49/184 vs daily 191/559, *p* = 0.02).

### Primary outcomes

The primary outcome of the study was the reduction of hospital antibiotic consumption. In 2018, year of the daily service of ID-cons, as compared to 2017, during which weekly consultations were carried out, antibiotic consumption decreased from 64 DDD/100bd to 60 DDD/100bd (*p* = 0.07).

In order to include the hospital units where the highest number of ID-cons were performed a sensitivity analysis was performed by excluding those units where less than 25th percentile of the ID-cons was performed (Oncology, Obstetrics/gynaecology and Psychiatry). Results from the sensitivity analysis showed that antibiotic consumption decreased from 67 DDD/100bd to 64 DDD/100bd (*p* = 0.01).

**Fig. 2:**
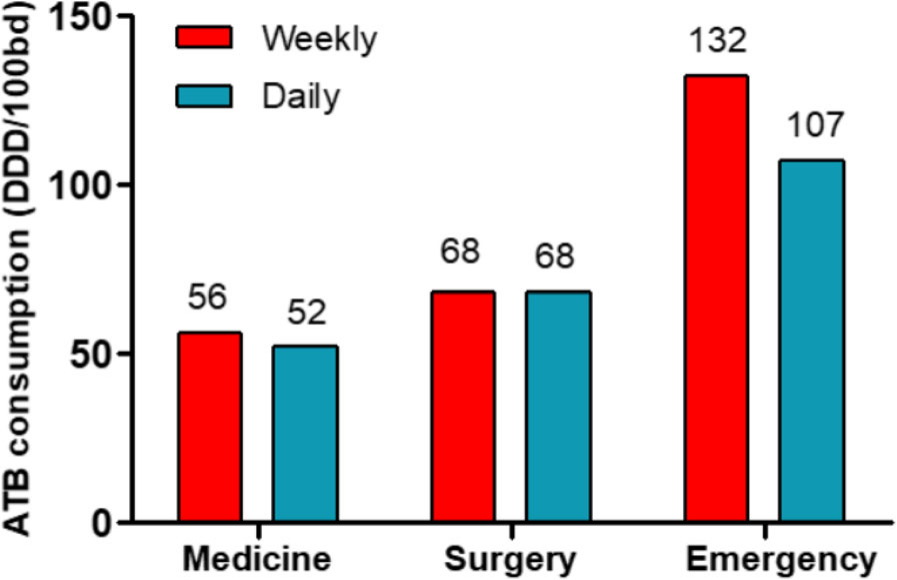
Antibiotic consumption expressed as DDD per 100 bed-days by department. *ATB* antibiotics; *DDD* Defined Daily Dose

Analysing the data by department, we obtained a small consumption reduction in the Medical Department (weekly 56 DDD/100bd vs daily 52 DDD/100bd), while consumption wasn’t reduced in the surgical department (weekly 68 DDD/100bd vs daily 68 DDD/100bd). The greatest reduction was observed in the Emergency Department where we observed a 19% reduction in the ATB consumption (weekly 132 DDD/100bd vs daily 107 DDD/100bd, *p* = 0.07) (Fig. 2). Focusing on the ICU, ATB consumption decreased from 131 DDD/100bd to 103 DDD/100bd (*p* = 0.007).

**Fig. 3:**
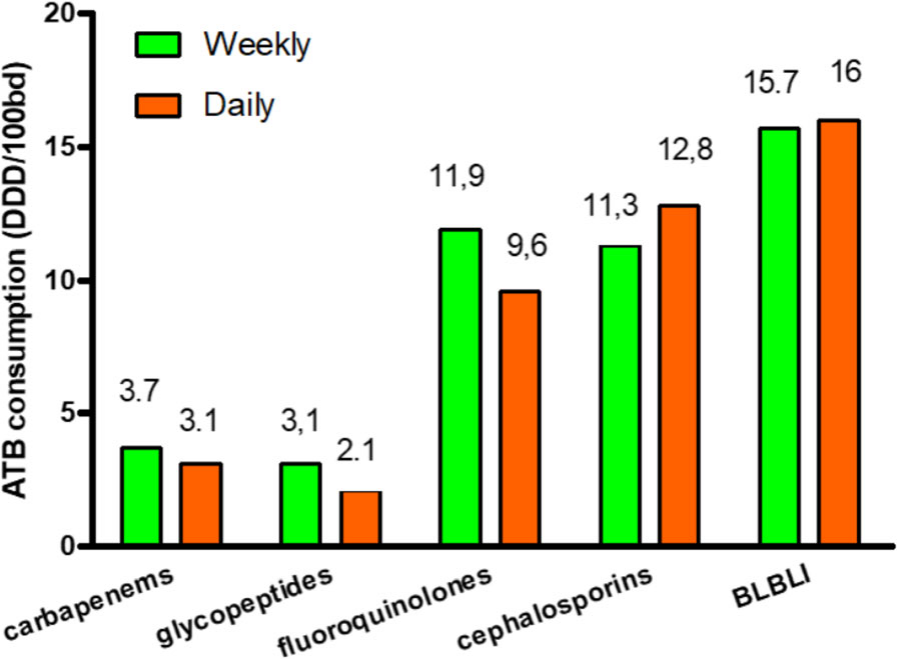
Antibiotic consumption by classes. *ATB* antibiotics; *BD* bed-days; *BLBLI* beta-lactam/beta-lactamase inhibitors; *DDD* Defined Daily Dose

Clustering the data by antibiotic classes, we have obtained a 33% reduction in glycopeptides use (weekly 3.1 DDD/100bd vs daily 2.1 DDD/100bd, *p* = 0.04) and a 19% reduction in fluoroquinolones (FQs) use (weekly 11.9 DDD/100bd vs daily 9.6 DDD/100bd, *p* < 0.001). A reduction, even though non statistically significant, was obtained in carbapenems use (weekly 3.7 DDD/100bd vs daily 3.1 DDD/100bd, *p* = 0.3). The reduction in glycopeptides, FQs and carbapenems use was not accompanied by an increase in other ATB classes (Fig. 3).

### Secondary outcome

During the study period, no change in overall in-hospital mortality was observed (5.2% in 2017 vs 5.2% in 2018). Regarding sepsis-related mortality, a non statistically significant increase in mortality was recorded (2017 19.3% vs 20.9%, *p* = 0.7).

## Discussion

In our study, the availability of daily ID-consultations was associated with a global reduction in antibiotic consumption in the whole hospital in spite of a similar distribution of infections among the two time periods. This reduction was not accompanied by a worsening of clinical outcomes.

The major reduction in the use of ATB was observed in the Emergency Department, especially in the ICU. It is worth to note that this was the department with the greatest increase in the number of ID-cons. The reduction in ATB consumption obtained in the Emergency Department was mainly due to the ID-cons performed in the ICU. In fact, only a small part of the ID-cons was provided in the emergency room (34/2552 of all the ID-cons). However, we believe that these ID-cons, albeit in a small degree, had an impact on the primary outcome. In fact, part of these patients was later admitted to the hospital and the decision to defer antibiotics pending culture results may have contributed to the overall reduction in ATB consumption. As known, the ICU represent the settings where ID consultants are greatly needed because of the higher circulation of MDR, higher usage of broad-spectrum antibiotics and more difficult-to-treat infections. Indeed, in our study the daily availability of the ID-consultant resulted in a higher number of ID-cons requested by the clinicians and subsequently in a significant reduction in ATB use. This correlation strengths our finding on the impact of ID consultations.

These findings are concordant with several previous studies. ID intervention was proved to be effective in reducing antimicrobial use without affecting clinical outcome in a recent review by Pulcini et al. [11]. In other studies, ID intervention resulted associated with a decreased mortality [19, 20] and length of stay [21] and lowered healthcare-associated costs [19].

In our study, the use of glycopeptides and carbapenems was considerably reduced. These two classes often represent the only therapeutic weapon to treat severe infections due to MDR bacteria as methicillin-resistant *Staphylococcus aureus* (MRSA) and extended-spectrum beta-lactamases (ESBL) producing *Enterobacterales*. Therefore, the judicious use of these antibiotics is one of the main outcomes of several antimicrobial stewardship (AMS) programs as underlined in the Global Action Plan on AMR by WHO [22]. Moreover, As highlighted by Goff et al. in their recent review on AMS, the optimization of antibiotic use should pass through education and collaboration [23]. In our opinion, ID-cons represents a valid method to improve appropriate use of ATB using an educational approach, instead of a restrictive one, in collaboration with the non-ID physicians.

It is noteworthy that in our study we did not observe an increment in the use of other antibiotic classes as a consequence of the reduction of glycopeptides, carbapenems and FQs. The so-called “squeezing the balloon effect” is an undesirable side effect of several AMS programs where the reduced use of one agent is associated with increased use of another [24, 25].

Another class that was reduced in a significant way in our study was the class of FQs. Reducing FQs use is crucial for at least two reasons. On the one hand, FQs-resistance in *Enterobacterales* in Italy is above 40%, meaning that the use of these agents as empiric therapy is no longer justified. On the other, in the past few years have been published several warnings on the adverse effect of FQs have been reported in the literature. In 2018 the U.S. Food and Drug Administration (FDA) released a Drug Safety Communication in which it advises against FQs use due to the serious side effects that likely outweigh the clinical benefits [26]. Lastly, fluoroquinolones are strongly associated with *Clostridioides difficile*-associated diarrhea [27]. Thus, reducing their use can be associated with a reduction in the rate of *C. difficile* infections.

Concerning other outcomes, our intervention resulted in a more comprehensive management of the infected patient by the ID-consultant; in fact, in 2018 ID-cons were asked sooner after hospital admission and more antibiotic therapies were decided by the ID-consultant. The importance of the Infectious Diseases Specialist for the management of patients with severe infections has been clearly demonstrated in a recent meta-analysis by Vogel et al. [28]. In their study, they have evaluated the impact of ID-consultation on the management and outcomes of patients with *Staphylococcus aureus* bacteremia. Overall 30-day mortality, 90-day mortality and relapse risk were significantly reduced in the group of the ID-consultation with a relative risk of 0.53, 0.77 and 0.62 respectively [28].

Moreover, the quality of the antibiotic use was improved as witnessed by the increment in the rate of appropriateness of ATB prescriptions.

In the study, half of the infections were healthcare associated. However, it has to be considered that those are exclusively the infections for which the clinician asked for ID advice. In our opinion, this is due to the fact the HAIs are often more difficult to treat and clinicians are more confident in treating CAIs without seeking for ID advice. Therefore, the real incidence of HAIs in our facility is inferior to the one observed in the study.

The study has some limitations. Firstly, we did not evaluate all the prescriptions done in the hospitals but just the ones for whom ID-consultant was asked for advice. Hence, we could not evaluate properly the appropriateness of ATB use in the entire hospital. This is a direct consequence of the nature of the study and it is the reason why appropriateness is a process outcome instead of a primary outcome. Contrarily, the consumption of ATB was evaluated in the whole hospital including the prescriptions for which the ID-consultant did not have a direct impact resulting in an underestimation of the effect of the intervention. This was partly but not completely avoided by the use of the sub-analysis on the hospital units in which an adequate number of ID-cons was performed. A second limitation is that we did not evaluate the impact of the reduction in ATB consumption on the circulation of MDR. However, to observe a reduction in MDR circulation a longer time period is needed. Nonetheless, we could have evaluated the impact of the reduction of FQs use on the incidence of *C. difficile*. This represents a future direction for our research. Similarly, we did not evaluate the occurrence of adverse events, due to the lack of follow-up visits in the weekly period. In any case, even though this could be an interesting outcome, the incidence of adverse events was not the target of our study. Moreover, it has to be acknowledged that, unfortunately, in our hospital the Pharmacy Unit does not provide recommendations regarding ATB dosing and this service is provided by the ID consultant during consultancies. In our opinion, a multidisciplinary AMS team, also including ID-pharmacists, would result in a greater reduction of ATB consumption.

Another limitation of the study is represented by the observational design of the study with its intrinsic risk of biases.

Patients receiving antibiotics prior to hospital admission were included in the study but detailed information of these cases was not available for the analysis.

## Conclusions

In conclusion, Infectious Diseases consultation was effective in reducing antibiotic use in the whole hospital and in the Emergency Department in particular without affecting in-hospital and sepsis-related mortality. These findings further prove that ID-consultation is a valid tool for a successful large-scale AMS.

## Data Availability

The dataset used and analysed during the current study is available from the corresponding author on reasonable request.

## Abbreviations

ACCI: Age-adjusted Charlson comorbidity index
AMR: Antimicrobial resistance
AMS: Antimicrobial stewardship
ATB: Antibiotic
BD: Bed-days
CAI: Community-acquired infection
CNS: Central nervous system
DDD: Defined Daily Dose
ESBL: Extended-spectrum beta-lactamases
FDA: Food and Drug Administration
FQs: Fluoroquinolones
HAI: Healthcare-associated infection
HCV: Hepatitis C virus
HSC: San Carlo Borromeo Hospital
ICU: Intensive care Unit
ID-CONS: Infectious Diseases consultation
IQR: Interquartile range
MDR: Multidrug-resistant
MRSA: Methicillin-resistant *Staphylococcus aureus*
UTI: Urinary tract infection
WHO: World Health Organization
